# Causal relationship between spondylarthritis and stroke in a European population: a two sample Mendelian randomization study

**DOI:** 10.3389/fimmu.2023.1253986

**Published:** 2023-10-18

**Authors:** Luofei Zhang, Kefu Yu, Jiping Huo, Shenghui Mei, Zhigang Zhao, Bin Zhu

**Affiliations:** ^1^ Department of Pharmacy, Beijing Tiantan Hospital, Capital Medical University, Beijing, China; ^2^ Department of Clinical Pharmacology, College of Pharmaceutical Sciences, Capital Medical University, Beijing, China

**Keywords:** cardioembolic stroke, causality, Mendelian randomization, spondyloarthritis, stroke

## Abstract

**Background:**

Observational studies have found an increased risk of stroke in patients with spondyloarthritis, but the results are susceptible to reverse causality and confounders. Therefore, the study aimed to further explore the association between spondyloarthritis and different subtypes of stroke by using a two sample Mendelian randomization (MR) analysis.

**Methods:**

Genetic instrumental variables for spondyloarthritis were identified using summary level data from a genome-wide association study involving 201,581 people. Summary statistics from the Multiancestry Genome-wide Association Study of Stroke Consortium were used to obtain genetic data on stroke. There was no sample overlap between the exposure and outcome datasets. Inverse-variance weighted was considered the primary MR method for causal analysis. Heterogeneity, pleiotropy and sensitivity analyses were performed to ensure robustness, and single nucleotide polymorphism (SNP) with potential confounders was further screened in the PhenoScanner database to better evaluate the stability of our study.

**Results:**

One SNP (rs1065045) was excluded due to schizophrenia. After excluding SNP (rs1065045), results of the second MR analysis were slightly different from the first, which were considered as the final result: a significant positive causality between spondyloarthritis and cardioembolic stroke (OR=1.296, 95% CI:1.094-1.534, *p*=0.003); a possible positive causality between spondyloarthritis and any stroke (OR=1.082, 95% CI:1.016-1.152, *p*=0.013)/any ischemic stroke (OR=1.086, 95% CI:1.013-1.163, p=0.020); no significant/possible causality between spondyloarthritis and small vessel stroke (OR=1.168, 95% CI:0.993-1.375, *p*=0.061). Insufficient power may be one possible reason why a causality was not observed between spondyloarthritis in our study.

**Conclusions:**

This study suggests that the possible causative effects of spondyloarthritis predicted by genetics on stroke may be limited to any stroke, any ischemic stroke, and cardioembolic stroke, especially the last.

## Introduction

Stroke is a leading cause of death and serious long-term disability worldwide, which caused a high disease burden (1, 2). Diabetes, obesity, hypertension, and so on have been reported as significant risk factors for stroke (3). However, studies on these traditional risk factors cannot fully explain the continued increase in stroke risk, and it is necessary and meaningful to actively explore other risk factors for stroke (4). Recently, there has been growing interest in exploring the effects of various types of arthritis on stroke risk.

Spondyloarthritis is a broad category of arthritis including ankylosing spondylitis, psoriatic arthritis, reactive arthritis, with a global prevalence of 0.4% to 2%, which affects the axial bones, peripheral joints, and tendons (5, 6). Whose onset is less than 45 years old, but the younger the patient is, the more typical it is (6). Ankylosing spondylitis, psoriatic arthritis, and undifferentiated spondyloarthritis have been found to be potentially associated with an increased risk of stroke in several observational studies (7–10). However, the association between spondyloarthritis and stroke subtypes has not been comprehensively reported, nor has the relationship with stroke been explored in the context of spondyloarthritis as a whole. In addition, observational studies are not conclusive in determining causality due to the possibility of residual confusion and reverse causality (11). More robust evidence is needed to explore the causal link between spondyloarthritis and stroke.

At present, Mendelian randomization (MR) analysis has attracted the attention of scholars, because it can partially overcome the limitation of traditional research on confounding and reverse causality, so as to clarify the causal relationship between two events. Consequently, a two-sample MR study was performed in our research to investigate the causal relationship between spondyloarthritis and different subtypes of stroke.

## Materials and methods

### Study design and data sources

**Figure 1:**
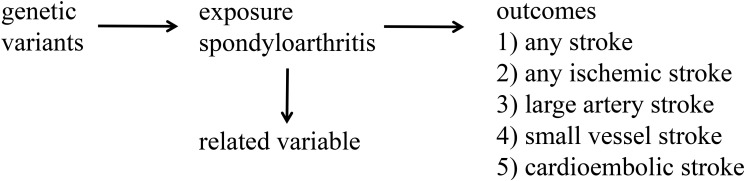
Directed acyclic graphs illustrating causal pathway from genetic variants to outcomes. Genetic variants influence the exposure, which has downstream effect on a related variable which does not affect the outcome. Related variables may be known or unknown, with known related variables referring to variables that have been reported to be associated with exposure but do not affect the outcome, and unknown related variables referring to variables that have not been reported to be associated with exposure but do not affect the outcome. There is no alternative pathway from the genetic variants to the outcome, the instrumental variable assumptions are satisfied.

A two-sample MR study based on genetic data designed to investigate a causality between spondylarthritis and strokes was conducted under three basic assumptions: (1) Genetic instrumental variations (IVs) are strongly associated with spondylarthritis; (2) Genetic IVs are not associated with total potential confounding factors; (3) Genetic IVs are not associated with stroke except by means of spondylarthritis (12). A directed acyclic graph illustrating the causal pathway from genetic variants to outcomes is shown in **Figure 1**. Participants in all original studies provided informed consent and approval for ethical review.

Characteristics of exposure/outcome genome-wide association studies (GWAS) used in this study are represented in **Supplementary Table 1**. The clinically diagnosed spondylarthritis was the exposure factor involved in this study. Phenotype (spondylarthritis) definitions were based on the International Statistical Classification of Diseases and Related Health Problems (ICD) coded hospital discharge or death. FinnGen data were used to extract genetic IVs, which combined imputed genotype data generated from newly collected and legacy samples from Finnish biobanks and digital health record data from Finnish health registries (13). All individuals volunteering to provide a biobank consent and to donate a biobank sample to a Finnish biobank were eligible for the FinnGen study Biobank recruitment (13). Summative data for spondylarthritis were obtained from a genome-wide analysis involving 16,380,342 single gene nucleotide polymorphisms (SNPs), including 3,037 cases and 198,544 controls of European descent (finn-b-SPONDYLOARTHRITIS, available at https://gwas.mrcieu.ac.uk/). Individual probe sets were assessed using the quality control (QC) metrics described in the Axiom™ Genotyping Solution Data Analysis User Guide (https://assets.thermofisher.com/TFS-Assets/LSG/manuals/axiom_genotyping_solution_analysis_guide.pdf) using the default thresholds, including call-rate ≥95% (13). Details regarding the participants, genotype platforms, and statistical analysis plan are available on the FinnGen website (https://www.finngen.fi/en).

The outcomes (stroke) dataset restricted to Europeans was obtained from the Multiancestry Genome-wide Association Study of Stroke (METASTROKE) consortium (14), consisting of 40,585 cases and 406,111 controls [n=40,585 any stroke (AS); 34,217 any ischemic stroke (AIS); 4,373 large artery stroke (LAS); 5,386 small vessel stroke (SVS); 7,193 cardioembolic stroke (CES)]. Participants of strokes were drawn from 17 studies with genome-wide genotypes imputed to 1000 Genomes Project (1000G) phase 1v3 or similar (14). The inclusion and exclusion criteria of the 17 studies were not completely consistent, and the common point was that patients were diagnosed with stroke according to the diagnostic criteria of the World Health Organization and stroke subtypes were classified according to TOAST (Trial of ORG 10172 in Acute Stroke Treatment) (14). Data on stroke included both first-ever stroke cases and recurrent stroke cases. The stroke studies all used imputed genotypes based on a multi-ancestral reference panel of at least 1000 G phase 1 and performed logistic regression analyses for five stroke traits (AS, AIS, LAS, CES, and SVS) (14). QC was performed for each study according to a standardized protocol, and specific information is available in Malik’s research (14). There was no participant overlap between the exposure and outcome datasets. The causal relationship between spondyloarthritis and five stroke traits (AS, AIS, LAS, CES, and SVS) was finally explored in this study.

### Genetic instrumental variable selection

All IVs were associated with spondylarthritis at a genome-wide significance level (*P* < 5 × 10^−8^) with linkage disequilibrium *r^2^* < 0.01 at a 10,000 kb window, confirming the independence of the selected IVs (15). The “harmonise_data” code was performed to harmonize alleles for exposure and outcome data. In addition, IVs with *F* values < 10 (calculated as *F =* (*R^2/^* (1-*R^2^*)) (n-2), *R^2 ^* = 2EAF(1-EAF) *β^2^*, n = simple size of exposure, EAF: effect allele frequency, and *β* was the estimated effect on arthritis) and SNPs with incompatible alleles were removed (16). When palindromic SNPs were existed, allele frequency information was used to infer the forward strand alleles (17). In order to satisfy the requirement that IVs were not associated with outcome, SNPs needed to be independent of stroke used in this study at a genome-wide level (*P* > 5×10^-5^).

### Statistical analysis

All statistics were conducted in R version 4.2.2, using the “TwoSampleMR” R package (version 0.5.6, Stephen Burgess, Chicago, IL, USA) for the two-sample MR analysis between exposure and outcome in our study. Inverse-variance weighted (IVW) method was adopted in the primary MR analyses and other four methods (MR-Egger, weighted median, simple mode, and weighted mode method) were used as sensitivity analyses. In addition, additional sensitivity analyses included heterogeneity test (Cochran’s Q-test), pleiotropic test (MR-Egger intercept test), and leave-one-out sensitivity analysis. The MR-Egger method enables the assessment of whether genetic variants have pleiotropic effects on outcomes, as well as providing consistent estimates of causal effects under weaker assumptions (18). The weighted median method can used to combine data from multiple genetic variants into a single causal estimate that is consistent even when up to 50% of the information comes from invalid IVs (19). The simple mode is a model-based assessment approach that offers pleiotropy robustness (20). For mode estimation, the weighted mode is sensitive to the difficult bandwidth selection (21). Multiplicative random effects model were used in the IVW method, as it can provide a more accurate estimation if there is any heterogeneity. To assess heterogeneity between SNPs in our study, Cochran’s Q-test was performed in IVW/MR-Egger analysis. Meanwhile, the MR-Egger intercept test was applied to detect horizontal pleiotropy. In addition, the leave-one-out sensitivity analysis was used to determine whether our assessment results were driven by specific SNPs with significant impacts. All statistical tests were two-tailed. In Cochran’s Q-test and MR-Egger intercept test, *P* values below 0.05 indicated statistical significance. The Bonferroni-corrected threshold of *p* < 0.01 (0.05/5 outcomes) was used to correct for multiple testing in the MR analysis. *P* values > 0.01 but less than 0.05 were considered suggestive evidence of a potential association (15). Scatter plots of SNP-related spondylarthritis and stroke risk were plotted using the data analysis function based on the MR platform to visualize the results of MR analysis. To maximize assurance that IVs were not associated with any potential confounders or risk factors for the outcome (stroke), further screening was conducted in the PhenoScanner database: if SNPs were associated with diabetes, obesity, hypertension, body weight, and so on, they were excluded (threshold of *p*-value = 1×10^-5^, *r^2 ^* = 0.8), and performed the MR analysis again (3, 22). The power online analysis platform (https://shiny.cnsgenomics.com/mRnd/) was used to calculate power for MR. Five data are required to calculate power, including 1) sample size (of outcome); 2) α (Type-I error rate, and the value of α was set to 0.05); 3) K (Proportion of cases in the study); 4) OR (True odds ratio of the outcome variable per standard deviation of the exposure variable); 5) R^2^_xz_ (Proportion of variance explained for the association between the SNP or allele score (Z) and the exposure variable (X)).

## Results

### The causal effect between spondylarthritis and different stroke

A total of 3 SNPs with *F* value more than 10 (minimum = 2858.3993, maximum = 8245.1238) were chosen as IVs for preliminary MR analysis. In addition, all of them were independent of strokes at a genome-wide level (*P* > 5×10^-5^) (**Supplementary Table 2**). Detailed characteristics of included SNPs with spondylarthritis are presented in **Table 1**.

**Table 1:** Characteristics of the included SNP loci associated with spondyloarthritis.

SNP	Position (hg19)	EA	OA	BETA	EAF	SE	*P*-value	*R* ^2^	*F* value
rs1065045	chr6:32611255	T	C	0.2130	0.2723	0.0324	4.78E-11	0.0180	3690.7461
rs10807943	chr7:5340664	C	T	-0.3449	0.9373	0.057	1.49E-09	0.0140	2858.3993
rs12190850	chr6:33864288	G	C	0.4662	0.1005	0.0469	2.80E-23	0.0393	8245.1238

EA, effect allele; OA, other allele; EAF, effect allele frequency; SE, standard error; R^2^ were calculated using the following formula: 2EAF(1-EAF) BETA^2^, where EAF is the effect allele frequency, BETA is the estimated effect on spondylarthritis, and SE is the standard error of the estimated effect; F value were calculated using the following formula: (R^2^/(1-R^2^)) (n-2), where R^2^ is the proportion of variance in spondylarthritis explained by each instrument and n is the sample size of the GWAS for the spondylarthritis association.

**Figure 2:**
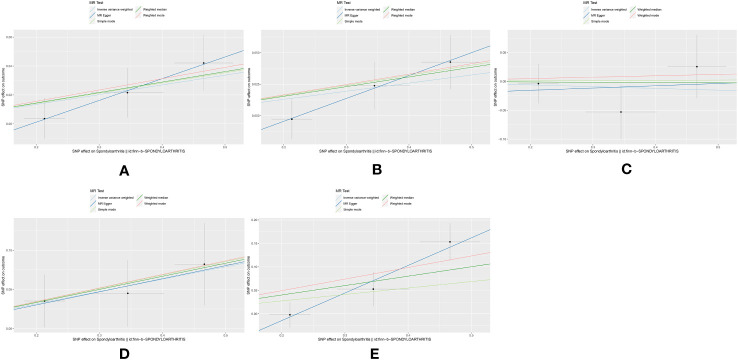
Scatter plots of the causality between exposure and outcome by five MR analyses. **(A)** Scatter plots of the causality between spondyloarthritis and AS by five MR analyses; **(B)** Scatter plots of the causality between spondyloarthritis and AIS by five MR analyses; **(C)** Scatter plots of the causality between spondyloarthritis and LAS by five MR analyses; **(D)** Scatter plots of the causality between spondyloarthritis and SVS by five MR analyses; **(E)** Scatter plots of the causality between spondyloarthritis and CES by five MR analyses.

The MR estimates and power analysis between spondyloarthritis and different strokes are presented in **Table 2**, and the visualized scatter plots of MR analysis are shown in **Figure 2**. Power was greater than 0.8 for all four stroke subtypes except LAS. In IVW analysis, four *P*-values were observed to range from 0.05 to 0.01, suggesting a suggestive causality between spondyloarthritis and the stroke. The possible causal associations were found with AS (OR=1.070, 95% CI:1.011-1.132, p=0.019), AIS (OR=1.067, 95% CI:1.002-1.136, p=0.042), SVS (OR=1.171, 95% CI:1.014-1.352, p=0.032), and CES (OR=1.222, 95% CI:1.014-1.472, p=0.036). In the weighted median analysis, the *P*-value of AS/AIS/SVS/CES was also less than 0.05, in which a significant causal effects was observed at CES (OR=1.222, 95% CI:1.055-1.416, p=0.008). However, there was no evidence to support a causal association between spondyloarthritis and LAS, which might be due to lack of power.

**Table 2:** Mendelian randomization estimates and power analysis between spondyloarthritis and strokes.

Outcome	SNP selection	N	IVW			MR-Egger			Weighted median			Simple mode			Weighted mode		
			OR (95% CI)	*P*	Power	OR (95% CI)	*P*	Power	OR (95% CI)	*P*	Power	OR (95% CI)	*P*	Power	OR (95% CI)	*P*	Power
**AS**	All	3	1.070 (1.011-1.132)	**0.019**	0.94	1.162 (0.968-1.395)	0.353	1.00	1.075 (1.010-1.144)	**0.024**	0.97	1.073 (0.987-1.167)	0.240	0.97	1.081 (1.004-1.165)	0.176	0.98
	Remove	2	1.082 (1.016-1.152)	**0.013**	0.95	NA	NA	NA	NA	NA	NA	NA	NA	NA	NA	NA	NA
**AIS**	All	3	1.067 (1.002-1.136)	**0.042**	0.88	1.200 (0.979-1.470)	0.330	1.00	1.080 (1.005-1.161)	**0.037**	0.96	1.083 (0.986-1.191)	0.238	0.97	1.086 (0.997-1.183)	0.199	0.98
	Remove	2	1.086 1.013-1.163)	**0.020**	0.90	NA	NA	NA	NA	NA	NA	NA	NA	NA	NA	NA	NA
**LAS**	All	3	0.970 (0.833-1.130)	0.697	0.08	1.040 (0.606-1.786)	0.910	0.11	0.995 (0.836-1.185)	0.957	0.05	1.005 (0.785-1.285)	0.974	0.05	1.025 (0.805-1.304)	0.862	0.07
	Remove	2	0.967 (0.790-1.183)	0.742	0.08	NA	NA	NA	NA	NA	NA	NA	NA	NA	NA	NA	NA
**SVS**	All	3	1.171 (1.014-1.352)	**0.032**	0.91	1.182 (0.743-1.879)	0.609	0.94	1.182 (1.013-1.378)	**0.033**	0.94	1.186 (0.972-1.449)	0.235	0.95	1.189 (0.992-1.425)	0.202	0.96
	Remove	2	1.168 (0.993-1.375)	0.061	0.81	NA	NA	NA	NA	NA	NA	NA	NA	NA	NA	NA	NA
**CES**	All	3	1.222 (1.014-1.472)	**0.036**	1.00	1.800 (1.254-2.582)	0.193	1.00	1.222 (1.055-1.416)	**0.008**	1.00	1.147 (0.890-1.478)	0.399	0.91	1.280 (1.002-1.635)	0.187	1.00
	Remove	2	1.296 (1.094-1.534)	**0.003**	1.00	NA	NA	NA	NA	NA	NA	NA	NA	NA	NA	NA	NA

NA, not available; SNP, single nucleotide polymorphism; AS, any stroke; AIS, any ischemic stroke; LAS, large artery stroke; SVS, small vessel stroke; CES, cardioembolic stroke; IVW, inverse-variance-weighted; N, the number of SNP; P value < 0.05 is indicated in bold.

### Assessment of sensitivity analyses

**Figure 3:**
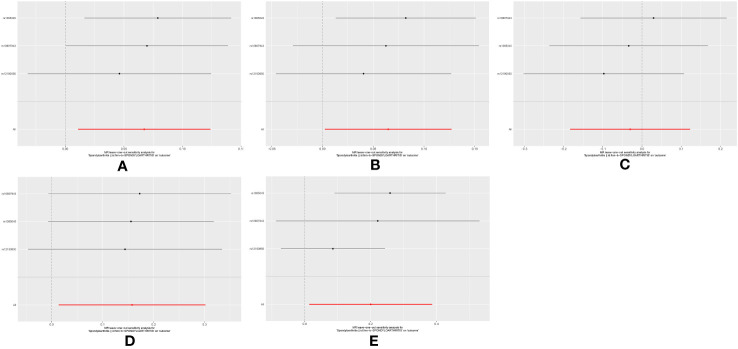
The leave-one-out sensitivity plots between exposure and outcomes. **(A)** The leave-one-out sensitivity plot between spondyloarthritis and AS; **(B)** The leave-one-out sensitivity plot between spondyloarthritis and AIS; **(C)** The leave-one-out sensitivity plot between spondyloarthritis and LAS; **(D)** The leave-one-out sensitivity plot between spondyloarthritis and SVS; **(E)** The leave-one-out sensitivity plot between spondyloarthritis and CES.

The results of heterogeneity and pleiotropy test are shown in **Table 3**. Among all heterogeneity/pleiotropy analyses, no *P* values were less than 0.05, suggesting that the results had a weak risk of bias and a high degree of reliability. The leave-one-out sensitivity analysis showed that the causality between spondyloarthritis and AS risk was driven by 1 SNP (rs12190850), the causality between spondyloarthritis and AIS/CES risk was driven by 2 SNPs (rs12190850 and rs10807943), and the causality between spondyloarthritis and SVS risk was driven by 3 SNPs (rs12190850, rs1065045 and rs10807943) (**Figure 3**). In addition, the associations were confirmed using sensitivity analyses including weighted median method (**Table 2**).

**Table 3:** Heterogeneity and pleiotropic tests of spondyloarthritis causally linked to strokes.

Exposure	Outcome	IVW		MR-Egger			
		Cochran’s Q	Q-*P* value	Cochran’s Q	Q-*P* value	Egger-intercept	Intercept-*P* value
Spondyloarthritis	Any stroke	0.8859	0.6421	0.0095	0.9223	-0.0288	0.5210
	Any ischemic stroke	1.4284	0.4896	0.0162	0.8987	-0.0409	0.4453
	Large artery stroke	1.3444	0.5106	1.2555	0.2625	-0.0233	0.8343
	Small vesell stroke	0.0779	0.9618	0.0763	0.7824	-0.0031	0.9740
	Cardioembolic stroke	5.2811	0.0713	0.3579	0.5497	-0.1319	0.2695

IVW, inverse-variance-weighted.

To further evaluate the stability of our study, the PhenoScanner database was used to exclude SNPs associated with any potential confounders, of which one SNP (rs1065045) was excluded due to schizophrenia (**Supplementary Table 3**). After removing the SNP (rs1065045), we performed the MR analysis again (**Table 2**). Heterogeneity detection found no heterogeneity in the second MR analysis (**Supplementary Table 4**). The estimated effects of spondyloarthritis on AS (OR=1.082, 95% CI:1.016-1.152, *p*=0.013), AIS (OR=1.086, 95% CI:1.013-1.163, *p*=0.020) and LAS were similar to those before. However, the estimated effects of spondyloarthritis on SVS and CES varied considerably. There was no evidence to support a causality between spondyloarthritis and SVS (OR=1.168, 95% CI:0.993-1.375, *p*=0.061), and a significant causal relationship was observed at CES (OR=1.296, 95% CI:1.094-1.534, *p*=0.003) by IVW analysis.

## Discussion

In this study, MR analyses were conducted twice to explore the causal effects between spondyloarthritis and different strokes, with slightly different results from the two MR analyses. Three IVs were included in the initial MR analysis. IVW analysis showed a suggestive positive causality between spondyloarthritis and AS/AIS/SVS/CES, but not LAS. Results were consistent with IVW except for a significant positive causal association between spondyloarthritis and CES rather than an implied positive causality in the weighted median analysis. This further verified the reliability of IVW results. Meanwhile, although no significant/suggestive *P*-value was found in other three MR analyses, the direction of effect of spondyloarthritis on different strokes (except LAS) was consistent among the five MR analyses methods. Additionally, heterogeneity and pleiotropy were not detected. Based on these results, IVW was used as the primary criterion for causation in the preliminary MR analysis.

Whereas traditional IVW methods can only give consistent estimates if all of the genetic variations in the analysis are valid IVs, weighted median analysis is used to combine data from multiple genetic variants into a single causal estimate that is consistent even when up to 50% of the information comes from invalid IVs (19). It was proved to have a better finite sample type 1 error rate than IVW (19). Due to the mechanism of the methodology itself, it is more difficult for weighted median analysis to obtain a statistically significant *P* value than IVW. However, when discussing the causality between spondyloarthritis and CES in preliminary MR analysis, the *P*-value of weighted median was significantly lower than that of IVW (0.008 vs 0.036). The weighted median result was significantly positive, while the IVW result was potentially positive, suggesting a confounding effect.

A statistically significant positive association between schizophrenia and stroke incidence risk was reported in a cohort study (n=363) (23), which was consistent in others (24–26). In the second MR analysis, the SNP (rs1065045) was excluded for schizophrenia. When SNP (rs1065045) was removed and MR analysis was performed again to explore the causality between spondyloarthritis and CES, IVW results showed a *P* value of 0.003 close to the weighted median *P* value in the initial MR analysis. This confirms the previous conjecture of confounding effects in the preliminary MR analysis. Furthermore, unlike the initial MR analysis (*P* = 0.032 by IVW), the second MR analysis (*P* = 0.061 by IVW) did not find a causal relationship between spondyloarthritis and SVS, which may also be caused by the removed SNP. Due to the limitation of the number of SNPs (n=2), the re-MR analysis could not obtain the results of other four MR methods except IVW, and pleiotropy test results were not available either. But the Cochran’s Q-test results in IVW suggest no heterogeneity, the IVW results from the second MR analysis were considered as the final results of the causal relationship between spondyloarthritis and stroke in this study.

A meta-analysis of 16 studies involving 18 cases (ankylosing spondylitis: 12 cases, psoriatic arthritis: 5 case, and undifferentiated spondyloarthritis: 1 case) by Kim et al. showed that spondyloarthritis patients had a 1.21 times greater risk of stroke than the general population (RR: 1.21; 95% CI: 1.0-1.47) (27). Fluctuating inflammatory activity is characteristic of spondyloarthritis, which may drive the increased stroke risk in spondyloarthritis (6). A case report found that early onset inflammatory activity may be at higher risk of adverse vascular events in patients with psoriatic arthritis (28). In addition, a study has shown that the increased cerebral blood flow is indirectly associated with atherosclerosis regarding persistent inflammation in patients with ankylosing spondylitis (29). Inflammatory activity in spondyloarthritis is followed by an increase in inflammatory cytokines at the joint site, which spill out into the circulation leading to adhesion of leukocyte and monocytes on the endothelial cells of the blood vessel wall, and then chemotaxis into the blood vessel wall, resulting in atherosclerosis (increased infiltration of inflammatory cells found in vulnerable plaques) and eventually vascular events such as stroke (30, 31). All the above evidences suggest that the positive causal relationship between spondyloarthritis and stroke may be mediated by inflammation, but more studies are needed to explore and confirm the mechanism of spondyloarthritis causal effect on stroke. Controlling inflammatory activity in spondyloarthritis patients may help reduce the risk of stroke, which also needs to be validated in large clinical trials. Biologics such as tumor necrosis factor-α have shown good anti-inflammatory effects in the treatment of spondyloarthritis (32). Although the causal relationship between spondyloarthritis and stroke has been explored in previous studies, the causality between spondyloarthritis and different stroke subtypes has not been investigated further.

There are several advantages in our study. Firstly, MR analysis can partially overcome the influence of mixed and reverse causality compared to traditional observational studies. In addition, the application of additional MR analysis methods, and the use of heterogeneity and level pleiotropy analyses enables the results’s robustness to be comprehensively evaluated. Finally, the study was rigorously designed to take into account the potential impact of SNPs with secondary phenotypic associations on the results, and the second MR analysis was performed to further assess the robustness of the findings.

Nonetheless, our study still has some limitations. First of all, the study population was limited to European ancestry, which means the conclusions may not apply to other races. Nevertheless, the GWAS database published so far is mainly of European descent, and it is hoped that GWAS data of other races can be available as soon as possible for further MR analysis. In addition, although various methods were applied to analyze and exclude confounders, potential confounders could not be completely removed. It must be acknowledged that the effects of “feedback mechanisms” and “cross-generation effects” mentioned in the literature were not examined by additional available statistical tests, implying that there is a risk of reverse causality through these mechanisms leading to bias in the MR analysis in this study (33). Fortunately, multiple analysis methods produced consistent results and found no evidence of horizontal pleiotropy or heterogeneity, confirming the findings of this study. What’s more, due to the limitation of the number of IVs, Mendelian Randomization Pleiotropy Residual Sum and Outlier analysis cannot be used in our study to test the horizontal pleiotropy. No pleiotropy was found in the initial MR analysis by MR-Egger intercept test, which ensured the reliability of the research conclusion. However, the results of leave-one-out analyses suggested that causality between exposure and outcome (AS/AIS/SVS/CES) may be driven by SNPs, which makes the reliability of the results questionable. Due to the fewer IVs, the second MR analysis could only obtain the results of IVW analysis, so the pleiotropy could not be evaluated; fortunately, there was no heterogeneity. In addition, there was no evidence in our results to support a causal relationship between spondyloarthritis and LAS, which might be due to lack of power. If larger scale GWAS data can be obtained in the future, it is necessary and meaningful to use MR analysis to further explore the causal relationship between spondyloarthritis and stroke.

## Conclusion

Genetic evidence of causal relationships between spondyloarthritis and strokes has been provided in our research. In conclusion, our study found a significant positive causality between spondyloarthritis and CES, and a potential positive causal relationship between spondyloarthritis and AS/AIS, while there was no evidence of a causal effect between spondyloarthritis and SVS. Furthermore, insufficient power may be one possible reason why a causality was not observed between spondyloarthritis and LAS in our study. It is important to acknowledge that the observed significant/potential causality between exposure and outcome may be driven by a single SNP. Through discussion, it was speculated that the association between spondyloarthritis and AS/AIS/CES might be mediated by inflammation. Suppressing inflammation may be a preventive strategy for AS/AIS/CES in spondyloarthritis patients, and well-designed clinical trials are needed to evaluate this.

## Data availability statement

Publicly available datasets were analyzed in this study. This data can be found here: data on spondyloarthritis (exposure) and stroke (outcome) are available separately at https://gwas.mrcieu.ac.uk/, https://megastroke.org/.

## Author contributions

LZ: Data curation, Formal Analysis, Methodology, Writing – original draft. KY: Data curation, Formal Analysis, Writing – review & editing. JH: Data curation, Writing – review & editing. SM: Data curation, Writing – review & editing. ZZ: Conceptualization, Methodology, Writing – review & editing. BZ: Conceptualization, Methodology, Writing – review & editing.
